# An Experimental Study on the Ductility and Flexural Toughness of Ultrahigh-Performance Concrete Beams Subjected to Bending

**DOI:** 10.3390/ma13102225

**Published:** 2020-05-12

**Authors:** In-Hwan Yang, Jihun Park, The Quang Bui, Kyoung-Chul Kim, Changbin Joh, Hyungbae Lee

**Affiliations:** 1 Department of Civil Engineering, Kunsan National University, Kunsan, Jeonbuk 54150, Korea; jhpark3@kunsan.ac.kr (J.P.); tqbui93@gmail.com (T.Q.B.); 2 Structural Engineering Research Institute, Korea Institute of Civil Engineering and Building Technology, Goyang, Gyeonggi 10223, Korea; kim6069@kict.re.kr (K.-C.K.); cjoh@kict.re.kr (C.J.); 3 Department of Environmental Management Business, EPS Engineering, Anyang, Gyeonggi 13930, Korea; leehb@epseng.com

**Keywords:** ultrahigh-performance concrete (UHPC), high-strength concrete (HSC), flexural behavior, steel fiber, ductility, flexural toughness

## Abstract

Ultrahigh-performance concrete (UHPC) and high-strength concrete (HSC) are currently widely used because of their distinct superior properties. Thus, a comprehensive comparison of the flexural behavior of UHPC and HSC beams is presented in this study. Nine UHPC beams and three HSC beams were subjected to pure bending tests to investigate the effect of various reinforcement ratios and steel fiber volume contents on the cracking and failure patterns, load-deflection behavior, ductility, and flexural toughness of these beams. The addition of steel fibers in the UHPC improved the energy absorption capacity of the beams, causing the UHPC beams to fail via rebar fracture. The deflection and curvature ductility indices were determined and compared in this study. The ductility indices of the HSC beam tended to decrease sharply as the rebar ratio increased, whereas those of the UHPC beam did not show a clear trend with respect to the rebar ratio. In addition, a comparison between the results in this study and the results from previous studies was performed. In this study, the addition of steel fiber contents up to 1.5% in UHPC increased the load capacity, ductility, and flexural toughness of the UHPC beams, whereas the addition of a steel fiber content of 2.0% did not significantly increase the ductility or flexural toughness of the UHPC beams.

## 1. Introduction

Ultrahigh-performance concrete (UHPC) and high-strength concrete (HSC) are advanced materials that are progressively gaining wider application in the construction industry [1,2,3,4]. UHPC and HSC can provide many advantages to satisfy some special or architectural requirements because of their high strength, abrasion resistance, durability, and low permeability. The differences between UHPC and HSC mix designs lead to distinct material properties and structural behaviors. Because of the elimination of coarse aggregates and the close packing of solid particles, UHPC exhibits a very dense microstructure, and the introduction of fibers improves the UHPC tensile strength [5,6].

The utilization of reinforcement bars (rebars) in the HSC matrix helps avoid brittle failures in HSC beams. However, as reported in some previous studies [7,8,9], increasing the number of rebars in HSC beams does not always improve the ductility of the beams. Rashid et al. [10] carried out bending tests on various reinforced concrete beams with a wide range of concrete strengths and reinforcement ratios. Their test results showed that the ductility of HSC beams decreased with increasing rebar ratio. Mohammadhassani et al. [11] conducted an experimental study on the failure modes of HSC beams with various rebar ratios and concluded that the HSC became more brittle with higher rebar ratios. In addition, increasing the rebar ratio led to lower ultimate deflections and a higher number of visible cracks in the beams.

Some studies [12,13,14,15,16] have been conducted to investigate the structural behavior of fiber-reinforced concrete and UHPC beams. In particular, the behavior of steel fiber-reinforced concrete beams under various loadings was investigated by Chalioris et al. [17,18,19]. Chalioris et al. [18,19] concluded that the addition of steel fibers in concrete could improve the strength and ductility of the reinforced concrete beams.

Yang et al. [13] also examined the influence of the concrete placing method on the flexural behavior of UHPC beams with a low rebar ratio. Su et al. [20] studied the mechanical behavior, failure mode, crack resistance, and ultimate load carrying capacity of UHPC beams. The addition of steel fibers in UHPC is a common method to enhance the tensile strength and control the cracking behavior of UHPC beams because steel fibers have a bridging effect on cracks in reinforced concrete beams [21,22,23,24,25]. Singh et al. [26] reported that the widening of the cracks was resisted by the steel fibers even after the rebar yielded, thereby increasing the load capacity of the beams. It is also expected that the ductility of UHPC beams can be improved by adding steel fibers. The effect of steel fibers on the ductility of UHPC beams was presented in the study of Yoo and Yoon [27]. Furthermore, according to the studies of Wu et al. [28], the addition of steel fibers to the UHPC mixture improved the energy absorption capacity of UHPC beams.

However, some researchers [21,29] presented contrary results. For example, Dancygier [21] reported that the ductility index of UHPC beams reinforced with steel fibers was 50%~80% less than that of conventional concrete beams. Huang et al. [29] also reported that the toughness of UHPC beams with 1.0% fiber volume content was greater than that of beams with 2.0% or 2.5% fiber volume contents. In particular, few studies have compared the ductility and toughness between UHPC and HSC beams. Therefore, an extensive comparison of the structural behavior between UHPC and HSC beams should be investigated.

Few design recommendations for UHPC members under flexure have been developed [1,30,31]. The design recommendations are rather limited. The formulation for calculating the flexural capacity in the existing recommendations does not have a closed form. Instead, it requires cross-section analysis with iterative routine based on the equilibrium condition and compatibility, which is accompanied by a predefined stress-strain relationship for UHPC and rebar. Therefore, to predict the flexural capacity of the UHPC beams using a practical method with a closed form, more accumulation of experimental data is necessary.

The objective of this study is to compare the flexural responses of HSC and UHPC beams regarding the cracking and failure patterns, load-deflection behavior, ductility index, and flexural toughness. The test variables include two types of concrete (i.e., HSC and UHPC), three different rebar ratios, and three different steel fiber contents. The HSC and UHPC beams were reinforced with rebar ratios of 0.79%, 1.18%, and 1.58%, and steel fiber volume contents of 1.0%, 1.5%, and 2.0% were used in the UHPC beams. Finally, the comparison in this study provides extensive information to understand the flexural behavior of HSC and UHPC beams.

## 2. Mix Design for UHPC and HSC 

**Figure 1:**
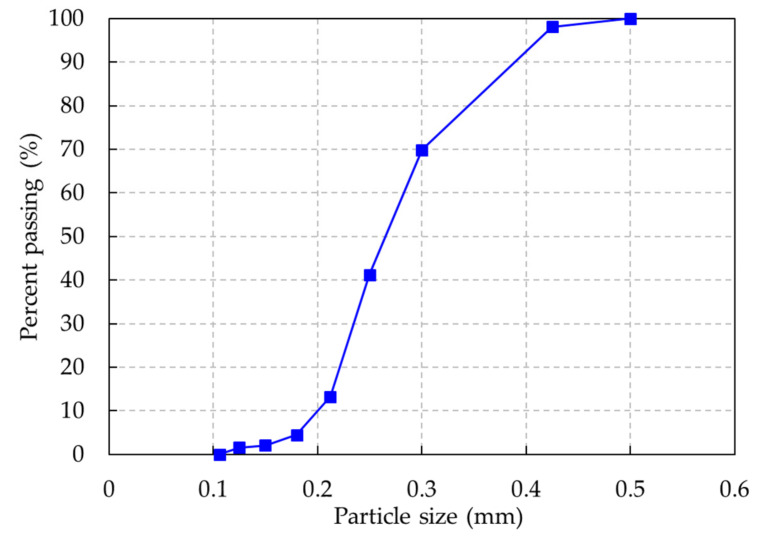
Particle size distribution of fine aggregate.

The target compressive strength of the UHPC and HSC specimens was 120 MPa, so the mixing proportions were designed to achieve the desired concrete strength. The mixing proportions of HSC and UHPC used in this study are presented in Table 1. Ordinary Portland cement (OPC) was used as the cementitious material for both HSC and UHPC mixtures. To obtain the strength requirement, the materials for each mixture were selected carefully. For the HSC mixture, coarse aggregates with a maximum size of 20 mm and a density of 2.6 g/cm^3^ were used, and the fine aggregates (Palma, Jeonju, Jeonbuk, Korea) were sands with diameters less than 0.5 mm. The particle size distribution of the fine aggregate is shown in Figure 1. The reaction between a pozzolanic material and calcium hydroxide, which is a product of hydration, densifies the microstructure of concrete and improves the strength of hardened cement-based materials, so silica fume was included in the HSC mixture. As a supplementary cementitious material, blast furnace slag, which could create a denser matrix and enhance the service life of concrete structures, was used in both HSC and UHPC mixtures. The physical and chemical properties of binder materials used in this study are presented in Table 2.

Some conditions were considered in this study, such as a low water-binder ratio, eliminating coarse aggregates, and packing solid particles. The low values of the water-binder ratio used for the UHPC mixture can reduce porosity, enabling a denser microstructure. However, the decrease in the water-binder ratio reduces the workability of fresh concrete. To achieve the workability and fluidity of concrete, a high-range water reducer admixture with a density of 1060 kg/m^3^ was used for the UHPC and HSC mixtures. The presence of coarse aggregates in the UHPC mixture could prevent the distribution of steel fibers in the concrete matrix and reduce the workability. In addition, the interface between the hydrated cement paste and the coarse aggregate is the weakest part in the material, and the bonding strength at this interface depends on the size of the coarse aggregates; failure usually occurs in this part of the hardened concrete [32]. Thus, coarse aggregates were not used in the UHPC mixtures in this study. To enhance the close packing of solid particles in the UHPC mixture, fine aggregates and filler materials were used. Crushed quartz with an average diameter of 10 µm and a density of 2600 kg/m^3^ was used as a filler material. Compared to silica fume, zirconium powder could improve the fluidity of the UHPC [33,34], and thus, it was chosen in the mixtures of UHPC. Straight steel fibers with a diameter of 0.2 mm and a length of 16.5 mm were included in the UHPC mixtures. The fibers had a density of 7500 kg/m^3^ and a yield strength of 2500 MPa. To investigate the effect of various steel fiber contents (measured as a percentage of the concrete volume), three different fiber contents were adopted: 1.0% (UHPC-F10), 1.5% (UHPC-F15), and 2.0% (UHPC-F20).

## 3. Material Properties

To obtain the compressive strengths of the HSC and UHPC, compressive tests were performed on cylindrical specimens with a diameter of 100 mm and a height of 200 mm. During the fabrication of the test beams, three cylindrical specimens were made for each batch and kept beside the beams to ensure the same curing conditions. The cylinders were tested with a compression testing machine under a maximum load capacity of 3000 kN, and the load was applied at a constant displacement rate of 0.8 mm/s until failure. To estimate the elastic modulus of the cylindrical specimens, three linear variable displacement transducers (LVDTs, CDP-100, Tokyo Sokki Kenkyujo, Tokyo, Japan), which measured the deformation of the specimen, were held by the upper ring and the lower ring.

The flexural tensile strength of HSC for the design of reinforced concrete structures is usually ignored, and thus, the flexural strength test for the HSC specimens was not performed in this study.

The measured compressive strength and elastic modulus of the HSC and UHPC specimens are presented in Table 3. The compressive strength results of the HSC and UHPC-F10 specimens were similar, and there was also no significant difference between the elastic modulus of the HSC and UHPC specimens. However, the elastic modulus of the UHPC-F20 specimens was slightly greater than that of the other specimens.

**Figure 2:**
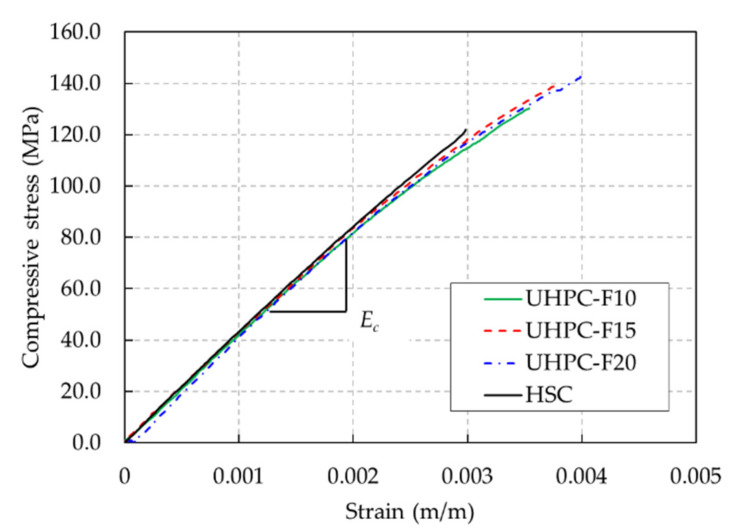
Compressive stress-strain curves of the HSC and UHPC mixtures.

The compressive strength of UHPC was improved by the addition of steel fibers up to a certain fiber content. The compressive strength of the UHPC-F15 specimens increased rapidly as the steel fiber content increased from 1.0% to 1.5%. However, the compressive strength of the UHPC-F20 specimens containing a steel fiber content of 2.0% was similar to that of the UHPC-F15 specimens containing a steel fiber content of 1.5%. The typical compressive stress-strain curves of the HSC and UHPC specimens are plotted in Figure 2. It shows that the elastic moduli of both the HSC and UHPC mixtures were similar, but the ultimate strains of the UHPC mixtures were greater than that of the HSC mixture.

UHPC is a remarkable material with a high tensile strength, and the presence of steel fibers in the concrete matrix leads to more ductile behavior. To determine the postcracking tensile behavior and the tensile strength of UHPC, a crack mouth opening displacement (CMOD) test was carried out using a notched prism under three-point loading. The prism specimen had a height of 100 mm, a width of 100 mm, and a length of 400 mm; a 10-mm-deep notch was cut into the tension face of this specimen. Based on the CMOD test results, the tensile strengths of the UHPC specimens were obtained by performing an inverse analysis.

The tensile strength test results in Table 3 show that the addition of steel fibers in UHPC improved the tensile strength of UHPC specimens. The tensile strength of UHPC increased with increasing steel fiber contents until reaching a certain limit. The tensile strength of the UHPC significantly increased as the steel fiber content increased from 1.0% (UHPC-F10) to 1.5% (UHPC-F15). However, the tensile strength of the UHPC specimens with 1.5% steel fiber contents (UHPC-F15) and that of the UHPC specimens with 2.0% steel fiber contents (UHPC-F20) were similar.

**Figure 3:**
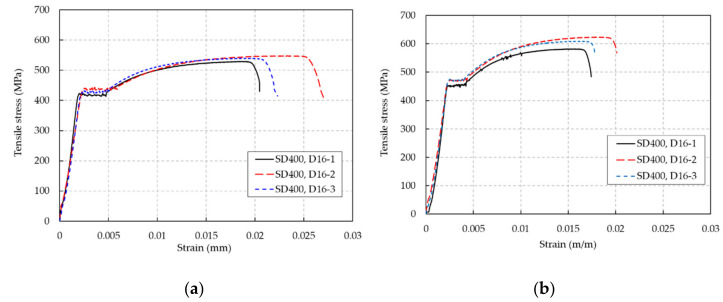
Tension test results of the rebars used. (**a**) Rebars used for the HSC beams, (**b**) rebars used for the UHPC beams.

The stress-strain curves of the rebars used for the HSC and UHPC beams are shown in Figure 3. In this study, the HSC and UHPC beams were reinforced by rebar with a nominal yielding strength of 400 MPa. The test results show that the mean yielding strength of 459.4 MPa of the rebar used for the UHPC beams was marginally higher than the mean yielding strength of 420.8 MPa of the rebar used for the HSC beams, as shown in Table 3. The mean ultimate strength and strain of the rebar used for the UHPC beams were 605.0 MPa and 0.018, while those of the rebar used for the HSC beams were 539.1 MPa and 0.023, respectively.

## 4. Experimental Program and Test Results

### 4.1. Details of the Test Beam and Instrumentation

**Figure 4:**
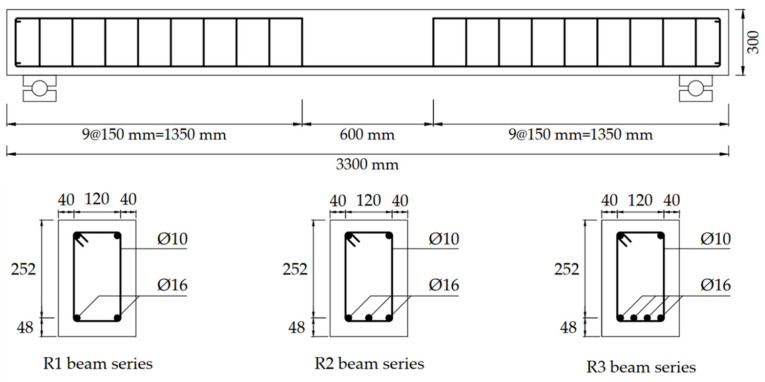
Dimensions of the beams.

To investigate and compare the flexural behavior of UHPC beams and HSC beams at a low reinforcement ratio, nine UHPC beams and three HSC beams were fabricated. The UHPC and HSC beams had the same dimensions with a length of 3,300 mm, a height of 300 mm, and a width of 200 mm, as shown in Figure 4. The details of the beam dimensions are presented in Table 3. The UHPC beams were designed to have a rebar ratio of less than 2.0% because the amount of reinforcement used in the UHPC beams is not high compared to the conventional concrete beams. Accordingly, the UHPC and HSC beams were designed with rebar ratios of 0.79%, 1.18%, and 1.58%, which was accomplished by utilizing 2, 3, and 4 rebars, respectively. The stirrups were provided outside the constant moment region to prevent shear failure. To determine the effect of thee fiber volume content on the flexural behavior of UHPC beams, fiber volume contents of 1.0%, 1.5%, and 2.0% were used for the beams.

**Figure 5:**
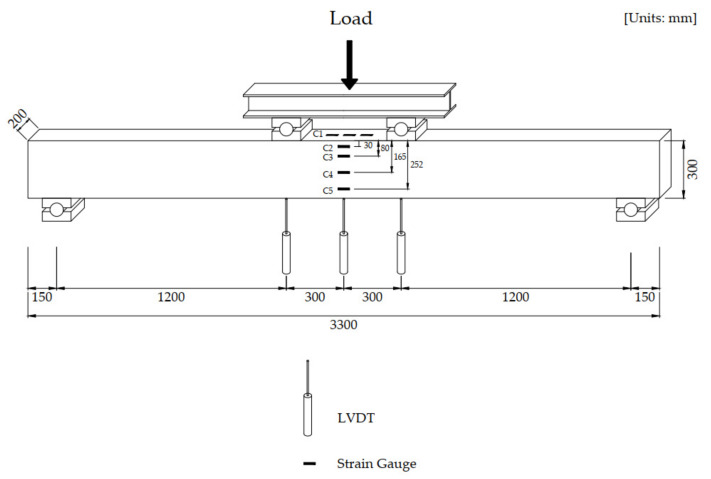
Instrumentation used for the flexural tests of the beams.

The four-point loading method was adopted for pure bending beam tests. The beam test setup is presented in Figure 5. The beams were placed on a pair of steel supports with a clear span of 3,000 mm, and the load was applied with a spreader beam. During the beam test, the deflection of each beam was obtained using three LVDTs located under the constant moment zone of the beam. To obtain the strain distribution along the beam depth, four strain gauges were attached on the side of the beam at the midspan and three strain gauges were attached on the top of the beam to obtain the compressive strain. To measure the strain in the rebars, four strain gauges were attached at the constant moment zone.

### 4.2. Test Results

#### 4.2.1. Effect of Steel Fiber Contents on the Cracking Pattern and Failure

**Figure 6:**
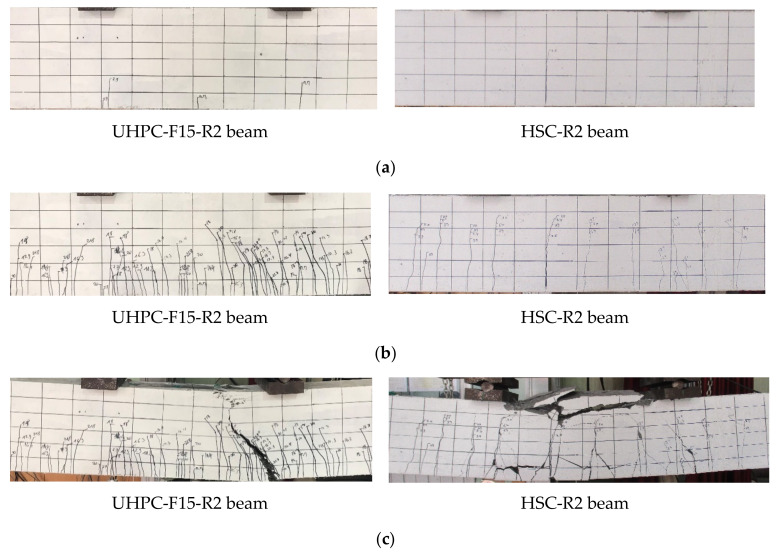
Typical cracking patterns of the UHPC and HSC beams. (**a**) Initial cracking state, (**b**) Yielding state, (**c**) Ultimate state.

Similar to the HSC beams, an initial crack in the UHPC beams was observed in the constant moment zone, as shown in Figure 6. For the UHPC beams, the initial cracks seem to be shorter than those in the HSC beams. In addition, the initial cracks in the HSC beams propagated more deeply upward from the bottom face to the top face of the beam with a length of approximately 150~200 mm. Moreover, the initial cracks observed in the UHPC beams were arrested with a shorter length of approximately 60~140 mm. This finding reveals that the UHPC beams exhibited better resistance to the initial crack propagation than the HSC beams.

**Figure 7:**
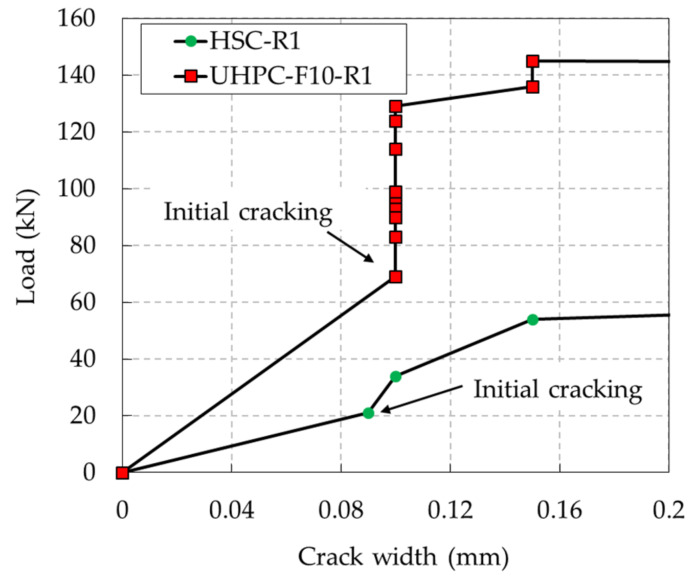
Comparison of the crack widths of the UHPC and HSC beams (up to a crack width of 0.2 mm).

**Figure 8:**
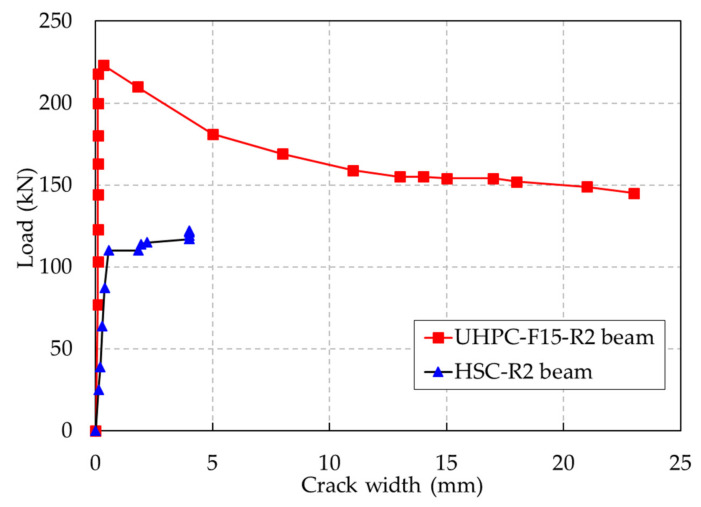
Comparison of the evolution of the crack width until failure of the UHPC and HSC beams.

The existence of steel fibers in the UHPC enhanced the flexural toughness in the initial loading stage of the UHPC beams; this evidence is illustrated in Figure 7. This figure shows that the width of the existing cracks in the HSC beams increased gradually, whereas the width of the existing cracks in the UHPC beams tended to remain constant until the rebars yielded. The reason for this phenomenon can be explained by the fact that in the HSC beams, only the rebar played a major role in carrying tensile stress across the cracks, whereas for the UHPC beams, the steel fibers transmitted the tensile stress into the matrix together with the rebar. In addition, the bonding strength of steel fibers in the concrete matrix inhibited crack widening in the later loading stages. Unlike the HSC beams, multiple hairline cracks were observed in the UHPC beams, whereas the HSC beams had a limited number of cracks in the later loading stages. Furthermore, the cracking spaces in the UHPC beams were smaller than those in the HSC beams due to the bridging effect provided by the steel fibers. In addition, the comparison of the evolution of the crack width until the failures of the UHPC and HSC beams is plotted in Figure 8. It shows that the major crack of the UHPC beam widened rapidly after the rebar yielded, while cracks of the HSC beams enlarged slowly until the ultimate state was reached.

**Figure 9:**
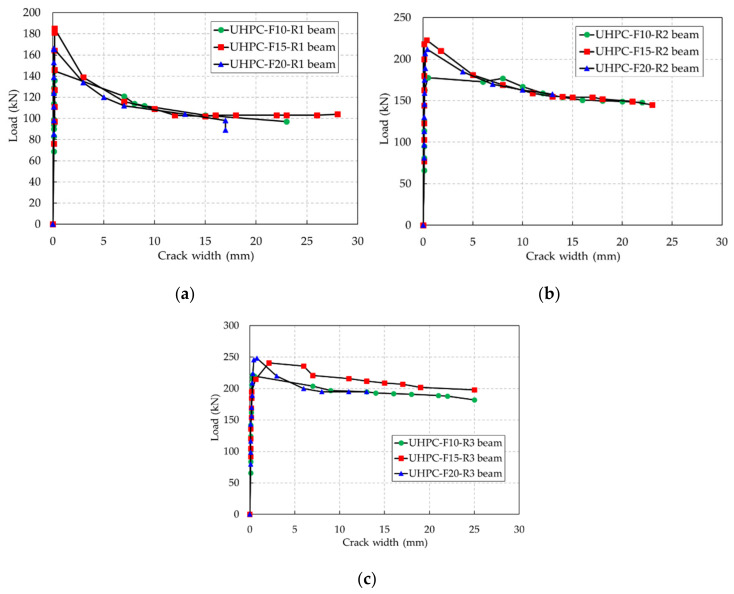
Effect of the steel fiber content on the evolution of the crack width. (**a**) Rebar ratio of 0.79%, (**b**) rebar ratio of 1.18%, (**c**) rebar ratio of 1.58%.

The effects of the steel fiber contents on the evolution of crack widths in the UHPC beams are shown in Figure 9. The evolution of the crack widths in the UHPC beams was less than 0.1 mm when loading was between the initial cracking and the formation of a major crack. However, the evolution of the crack widths was drastic after a major crack occurred. In addition, the crack widths at the descending part of loading were approximately the same for each beam, although the steel fiber contents were different.

**Figure 10:**
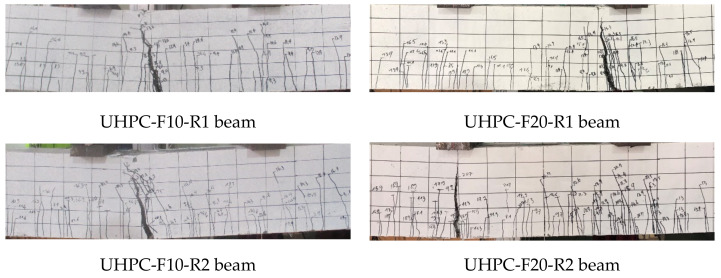

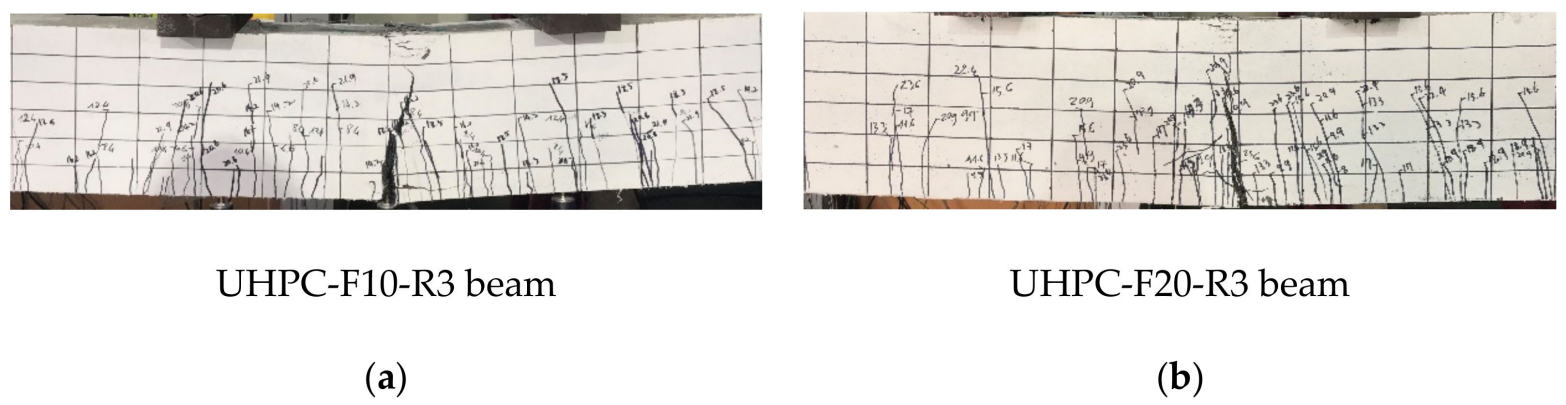
Typical failures of the UHPC beams. (**a**) 1.0% steel fiber content, (**b**) 2.0% steel fiber content.

**Figure 11:**
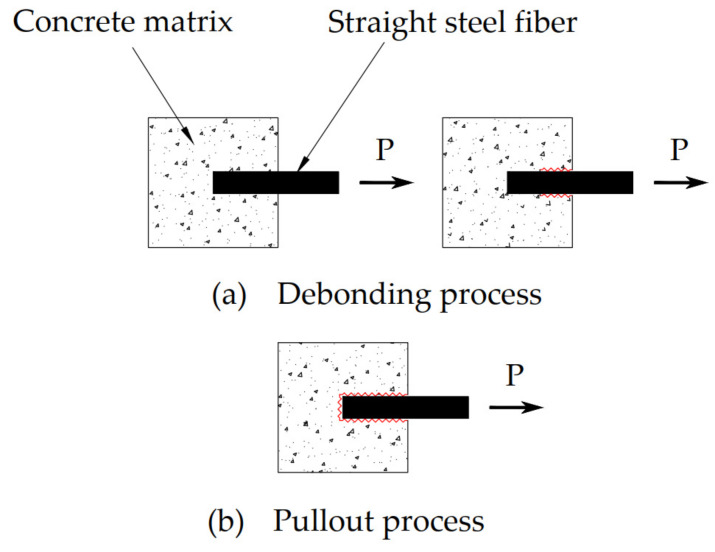
Pullout behavior of straight steel fibers.

The failure of the HSC beams occurred abruptly by concrete crushing in the compressive zone. In contrast, the UHPC beams failed due to a major crack that propagated upward to the compressive zone and widened remarkably after the rebar yielded, as shown in Figure 10. The width of the major crack in the UHPC beam was larger than that of the other cracks, whereas the cracks in the HSC beams had similar widths, as shown in Figure 6. For the UHPC beams, steel fibers played a role in bridging cracks, transmitting tensile stress and contributing to the prevention of brittle failure. Thus, the failure of UHPC beams did not occur suddenly but steadily. The failure of the UHPC beams is closely related to two main fracture processes at cracking localization, as shown in Figure 11, which are debonding on the interface between the steel fibers and matrix and the pullout of steel fibers from the matrix.

**Figure 12:**
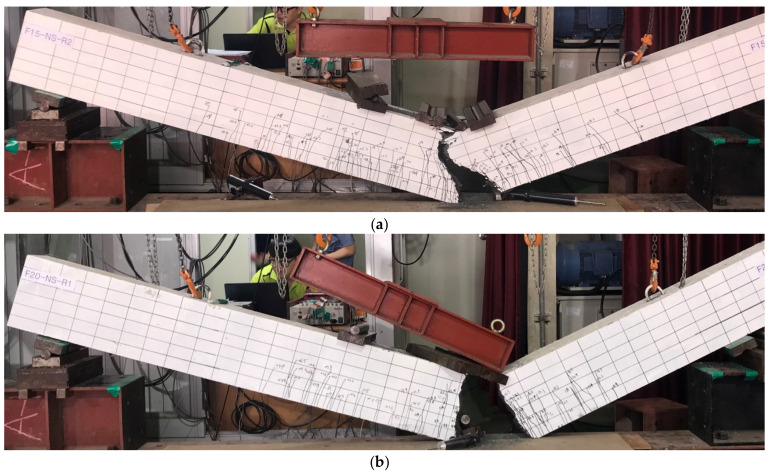
Failure of the UHPC beams by rebar fracture. (**a**) Failure of the UHPC-F15-R2 beam; (**b**) failure of the UHPC-F20-R1 beam.

In this study, it was interesting that the UHPC-F15-R2 and UHPC-F20-R1 beams collapsed from rebar fracture, as shown in Figure 12. This failure was due to the addition of steel fibers in the concrete matrix, which improved the energy absorption capacity of the beams. When the steel fibers were pulled out of the matrix, the substantial energy absorbed by the fibers was released. This energy release led to the fracture of the rebar and the subsequent beam failure.

#### 4.2.2. Load-Deflection Behavior of the UHPC and HSC Beams

**Figure 13:**
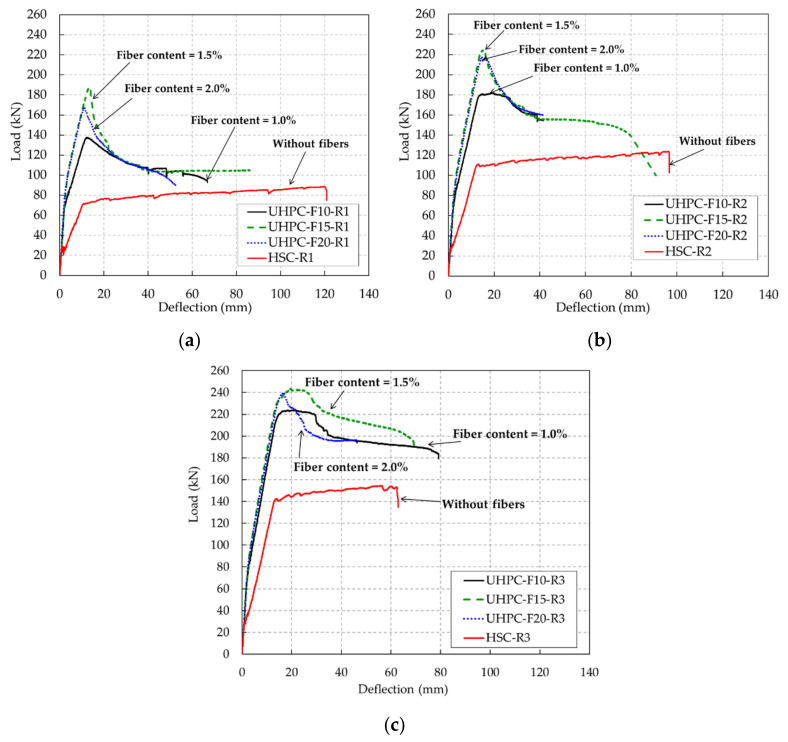
Comparison of the load-deflection relationships of the UHPC and HSC beams. (**a**) R1 series beams (rebar ratio of 0.79%), (**b**) R2 series beams (rebar ratio of 1.18%), (**c**) R3 series beams (rebar ratio of 1.58%).

A comparison of the load-deflection behavior of the UHPC and HSC beams is shown in Figure 13. This figure shows that the load-deflection behavior of the HSC and UHPC beams was different. This difference existed because of the distinct mechanical properties of HSC and UHPC, which gave the beams different flexural behavior. After initial cracking, the UHPC beams were stiffer than the HSC beams because the UHPC beams contained steel fibers. The steel fibers in the UHPC resisted crack development in the initial cracking stage, as mentioned in the previous section; thus, the UHPC beams became stiffer than the HSC beams.

After the rebars yielded, the UHPC beams and HSC beams exhibited distinct trends. The load-deflection curves of the UHPC beams after rebar yielding exhibited a descending part, whereas those of the HSC beams exhibited a slight ascending part. It can be assumed that the ascending part of the HSC beams was attributed to the strain hardening of the rebar, which carried the tensile stress of the beam after the initial crack formed. However, for the UHPC beams, the major crack gradually widened and led to the pullout of steel fibers, which resulted in a decrease in the resisting capacity of the UHPC beams.

Compared to the HSC beam at the same rebar ratios, the UHPC beams always exhibited a superior flexural strength because of the denser microstructure of the matrix and the presence of steel fibers in UHPC. For a rebar ratio of 0.79%, the maximum load of the UHPC-F10-R1 beam was 137.6 kN, which was 1.5 times greater than that of the HSC-R1 beam. Similarly, at rebar ratios of 1.18% and 1.58%, the UHPC beams with various steel fiber contents also presented a greater maximum load capacity than the HSC beams. The HSC and UHPC mixtures in this study were designed to obtain the same target compressive strength of 120 MPa, and thus, the strength properties of the UHPC mixture did not show a significant improvement compared to those of the HSC mixture. Meanwhile, this study focused on the benefit of the use of UHPC with regards to the structural behavior. In particular, according to test results of this study, the flexural capacity of the UHPC beams exceeded 1.5 times that of the HCS beams. This means that the structural designers could apply a smaller cross-section, for which the strength of the member remains while reducing the weight of the structures. Accordingly, it could also reduce the size of the foundation, which decreases the construction cost and saves the materials. In addition, at the service stage, UHPC beams demonstrated robust cracking control when compared to HSC beams.

However, it is interesting to note that the maximum load of the UHPC beam with 1.5% steel fiber content was greater than that of the UHPC beam with 2.0% steel fiber content. This phenomenon was also observed at rebar ratios of 1.18% and 1.58%. This phenomenon might be caused by the uneven distribution of steel fibers in the concrete matrix with high volume contents.

The deflection at the ultimate stage of the UHPC beams did not show a clear tendency as the rebar ratio increased, whereas the deflection at the ultimate stage of the HSC beams tended to decrease with increasing rebar ratio. Furthermore, the deflections at the ultimate stage of the UHPC beams at a rebar ratio of 0.79% were smaller than the deflections at the ultimate stage of the HSC beam. However, the deflections at the ultimate stage of the UHPC beams at a rebar ratio of 1.58% were more similar to the deflection at the ultimate stage of the HSC beam. This finding implies that at a higher rebar ratio, the UHPC beams had higher ductility than the HSC beams.

**Figure 14:**
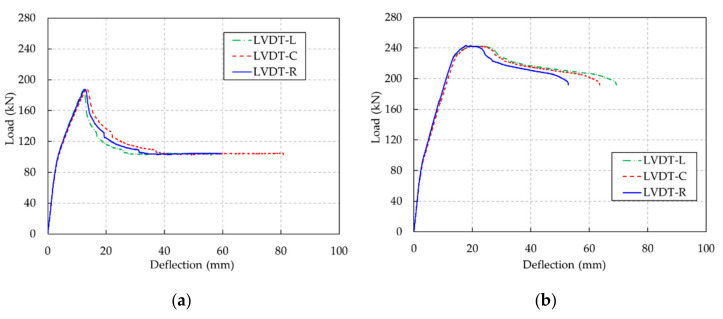
Load-deflection curves obtained by three different LVDTs. (**a**) UHPC-F15-R1 beam, (**b**) UHPC-F15-R3 beam.

Moreover, the load-deflection curves obtained by three LVDTs at the midspan of the beams are shown in Figure 14. Before a major crack occurred, the deflections by three LVDTs were approximately the same. However, after a major crack occurred, displacement measurements exhibited three different deflections at the same load. This phenomenon was due to the major crack, namely, cracking localization at the constant moment zone, affecting the curvature as well as the deflection of the beams [21].

#### 4.2.3. Effect of Rebar Ratios and Fiber Contents on the Ductility of the UHPC and HSC Beams

**Figure 15:**
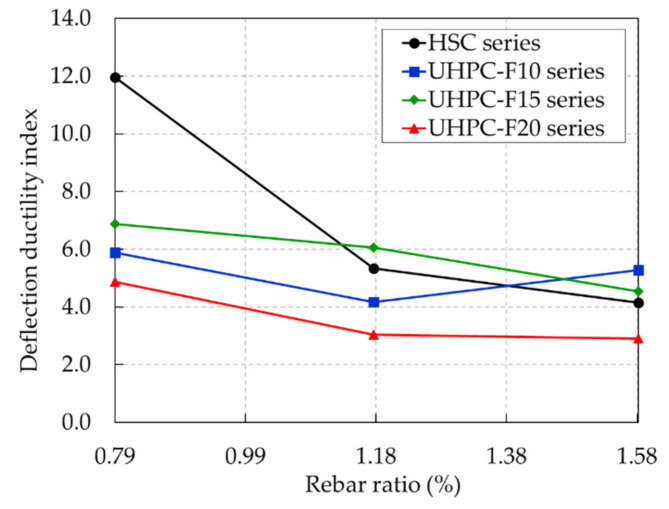
Deflection ductility indices of the UHPC and HSC beams with various rebar ratios.

The ductility index is defined as the ability of a structural member to resist a large deformation after the elastic stage without a sudden drop in strength. In this study, the deflection ductility index was estimated as the ratio between the ultimate deflection and the deflection in the rebar yielding stage. Figure 15 shows the deflection ductility indices of the UHPC beams and HSC beams with various rebar ratios; these results are also summarized in Table 4.

For a rebar ratio of 0.79%, the UHPC beams exhibited much smaller deflection ductility than the HSC beam. The deflection ductility indices of the UHPC-F10-R1, UHPC-F15-R2, and UHPC-F20-R3 were 5.9, 6.9, and 4.9, respectively, whereas the deflection ductility index of the HSC-R1 beam was 12.0. This large deflection ductility index of the HSC-R1 beam was due to the large deflection at the ultimate state of the HSC beams observed in Figure 13, which caused ductile characteristics in the HSC beams at a low rebar ratio.

**Figure 16:**
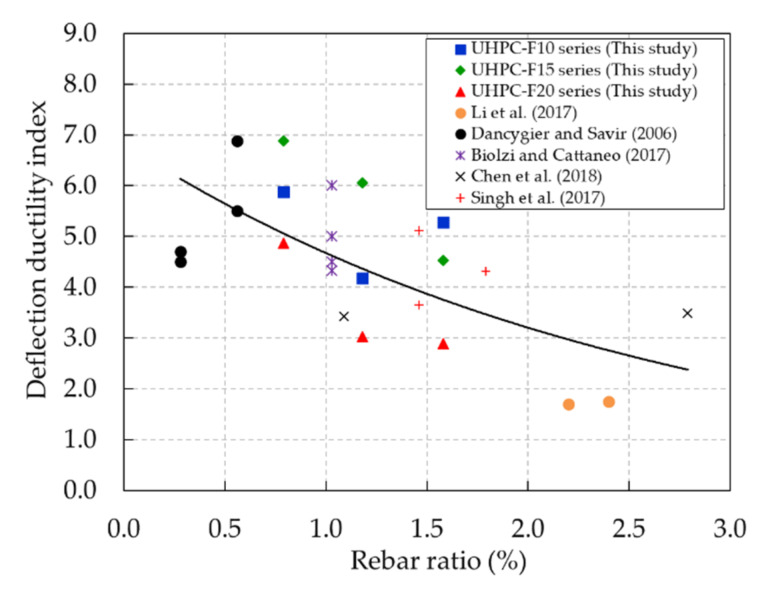
Comparison of the experimental results of the deflection ductility in this study and in previous studies.

The test results show that the deflection ductility of the HSC beams dropped dramatically with increasing rebar ratios; this phenomenon was also observed in a previous study [8]. The deflection ductility of the UHPC beams did not exhibit a clear tendency with an increasing rebar ratio. The deflection ductility indices of the UHPC-F15 and UHPC-F20 beam series decreased consistently with an increase in the rebar ratio. On the other hand, the deflection ductility of the UHPC-F10 beam series decreased slightly as the rebar ratio increased from 0.79% to 1.18% and then increased as the rebar ratio increased from 1.18% to 1.58%. Thus, a comparison between the results in this study and the results from previous studies [16,26,35,36,37] is shown in Figure 16. This figure shows that the overall deflection ductility of the UHPC beams decreased with an increasing rebar ratio. This result indicates that the deflection ductility of UHPC and HSC beams has a similar tendency as the rebar ratio increases.

The influence of the steel fiber content on the deflection ductility indices of the UHPC beams can also be found in Figure 15. The deflection ductility indices of the UHPC-F15 beam series were always greater than those of the other UHPC beam series with the same rebar ratio. At a rebar ratio of 0.79%, the UHPC-F15-R1 beam had the greatest deflection ductility index of 6.9, whereas the deflection ductility indices of the UHPC-F10-R1 and UHPC-F20-R1 beams were 5.9 and 4.9, respectively. Similarly, at a rebar ratio of 1.18%, the deflection ductility index of the UHPC-F15-R2 beam was greater than those of the UHPC-F10-R2 and UHPC-F20-R2 beams. However, at a rebar ratio of 1.58%, the UHPC-F10-R3 beam exhibited a greater deflection ductility index than the other beams; the UHPC-F20-R3 beam had the lowest deflection ductility index. Test results showed that increasing the steel fiber content could improve the deflection ductility of the UHPC beams until reaching a certain limit (1.5% in this study). Finally, this phenomenon might be affected by the distribution and orientation of steel fibers in the concrete matrix because the steel fiber contents did not consistently improve the deflection ductility of the UHPC beams.

**Figure 17:**
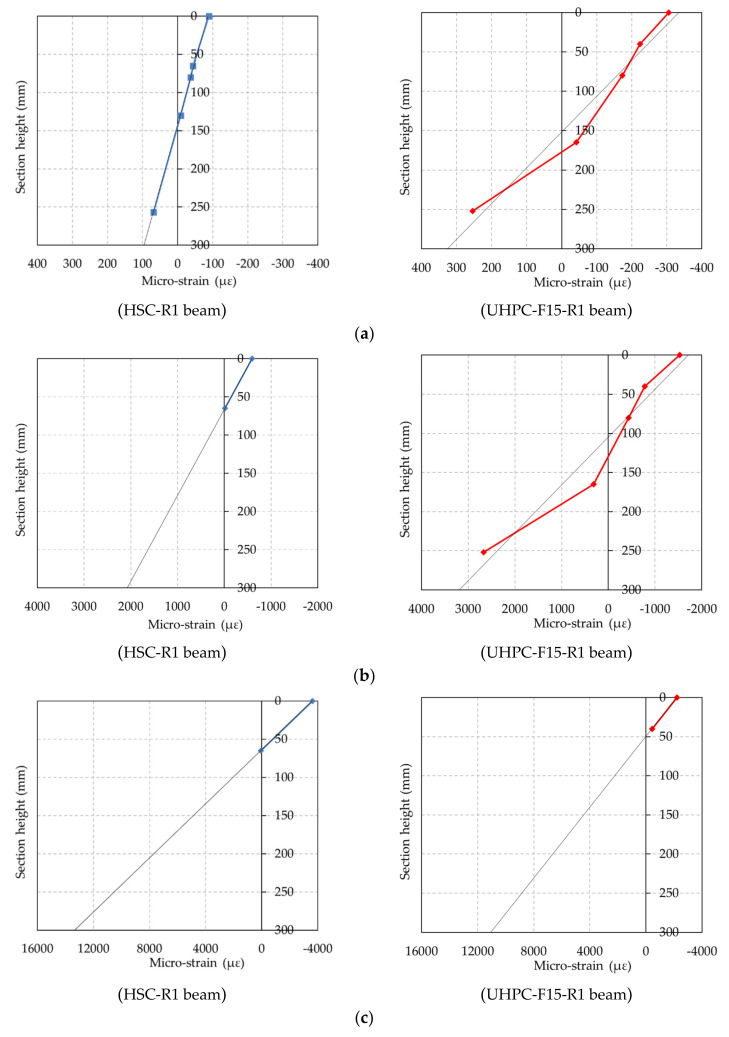
Strain distributions of the UHPC and HSC beams. (**a**) Initial cracking stage, (**b**) Rebar yielding stage, (**c**) Ultimate stage.

The strain distribution along the beam depth of both UHPC and HSC beams was measured during the beam test. Because of the propagation of cracks during loading, some strain gauges on the surface of concrete beams were destroyed. Thus, the linear strain distribution from extrapolation by using the measurements was assumed. The typical strain distributions at the initial cracking, rebar yielding, and ultimate stages of the UHPC and HSC beams are illustrated in Figure 17.

The test results show that the strains at the extreme compression and tension fibers at the constant bending region increased as the load increased. Compared to the HSC beam, the strains at the compression and tension fibers of the UHPC beam were greater at the initial and rebar yielding stages. These results imply that the flexural capacity at the cracking and rebar yielding stage of the UHPC beams is greater than that of the HSC. Due to the presence of steel fiber in the UHPC, it resisted the widening of the cracks and delayed the cracking and yielding stage, resulting in the greater strains at the compression and tension fibers of the beams. 

Moreover, at the ultimate stage, the strain at the top fiber of the HSC beams nearly reached the ultimate strain of concrete, whereas the strain at the top fiber of the UHPC beam was less than the ultimate strain of concrete. This implies that the crushing of concrete at the top fiber for the HSC beams would occur at the beam failure, whereas the crushing of concrete at the top fiber for the UHPC beams would not occur at the beam failure. This rationale can be supported by the actual beam failure patterns shown in Figure 6.

**Figure 18:**
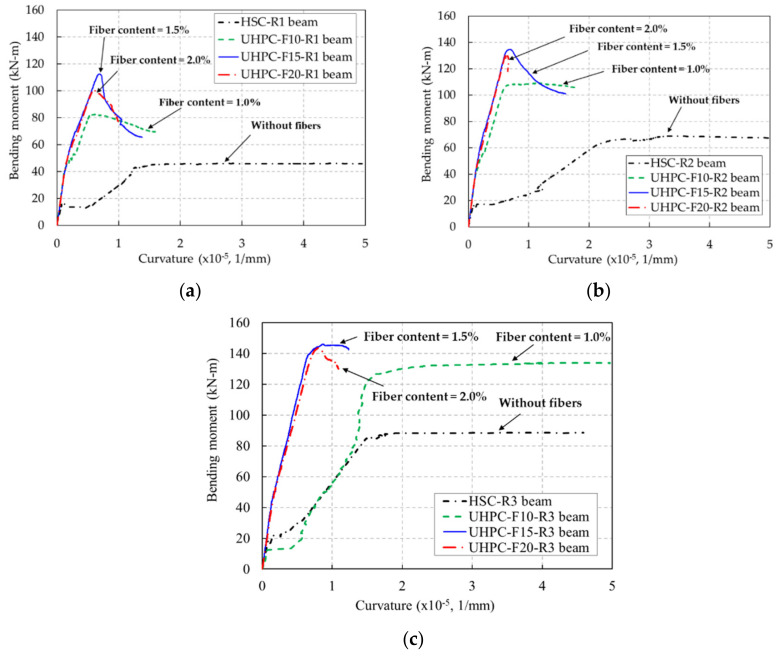
Moment-curvature curves of the UHPC and HSC beams. (**a**) R1 series beams (rebar ratio of 0.79%), (**b**) R2 series beams (rebar ratio of 1.18%), (**c**) R2 series beams (rebar ratio of 1.58%).

The moment-curvature curves of the UHPC and HSC beams are illustrated in Figure 18. The curvature of each beam was calculated using strain measurements on the side of the beam at the midspan and strain measurements on the top of the beam. Because of the destruction of the strain gauges due to cracking, the curvatures of several beams were not obtained until the ultimate stage. 

After initial cracking, compared to the HSC beams, the slopes of the moment-curvature curves of the UHPC beams were steeper. This means that the flexural rigidity of the UHPC beams was greater than that of the HSC beams. These results also indicate that the presence of steel fibers in the UHPC matrix prevented the widening of cracks and contributed to the development of flexural rigidity of the UHPC beams.

In addition, the curvature ductility indices of the UHPC and HSC beams were also examined. The curvature ductility indices of the UHPC and HSC beams were determined by the ratio of the curvature at the ultimate stage to the curvature at the rebar yielding stage. After the cracking of concrete in the tension region, the values of the measurement of the strain on the concrete surface were not reliable. Accordingly, the curvature of the beam at the ultimate stage was obtained from the strain distribution along the beam depth, which was extrapolated based on the measured values of strain, as shown in Figure 17.

**Figure 19:**
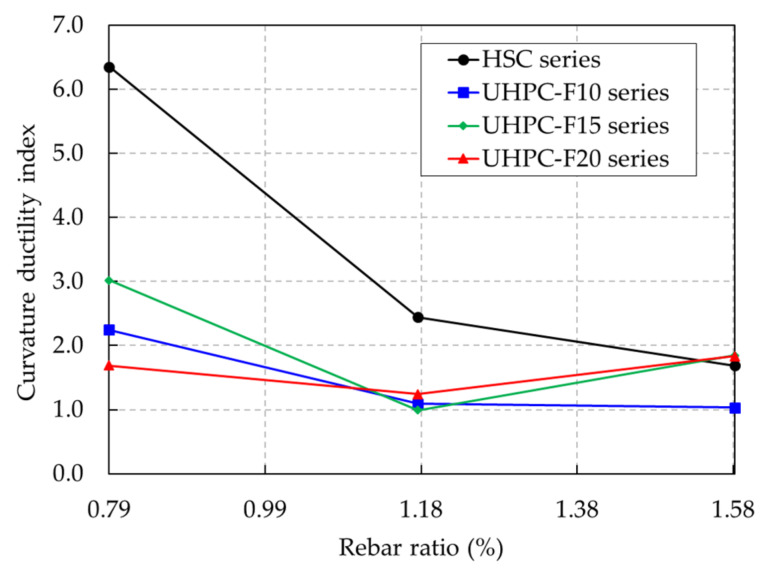
Curvature ductility indices of the UHPC and HSC beams with various rebar ratios.

The curvature ductility indices of the beams are shown in Figure 19 and listed in Table 4. The curvature ductility of the UHPC and HSC beams exhibited a reducing tendency as the rebar ratio increased. It was similar to the deflection ductility tendency shown in Figure 15. The HSC beam presented the greatest curvature ductility at a rebar ratio of 0.79% but it decreased dramatically at the high rebar ratio. The overall curvature ductility of the UHPC beams presented a gradual decrease with increasing rebar ratios. 

The curvature ductility indices of the UHPC beam series with 1.5% steel fiber content were greater than those of the other UHPC beam series at the rebar ratios of 0.79% and 1.18%. Therefore, test results showed that increasing the steel fiber content could improve the curvature ductility of the UHPC beams until reaching a certain limit (1.5% in this study).

#### 4.2.4. Flexural Toughness of the UHPC and HSC Beams

To evaluate the energy absorption capacity of the UHPC beams, the flexural toughness of each beam was estimated. The flexural toughness of the fiber-reinforced concrete beam can be calculated using several methodologies, which are reported in a study by Aslani and Samali [38]. In this study, the flexural toughness of each beam was obtained by calculating the area under the load-deflection curves shown in Figure 13. The flexural toughness values of the UHPC and HSC beams are given in Table 5. The UHPC and HSC beams were reinforced with a normal strength rebar with a nominal diameter of 16 mm (D16). The rebars used for the UHPC and HSC beams had yield strength values of 459.4 and 420.8 MPa, respectively, as shown in Table 3. Because the differences in the yield strength values of the adopted rebars in each beam affect the evaluation of the flexural toughness, a normalization is performed using the following equation.

(1) $$FT_{normalized}=FT\times \frac{f_{y,HSC}}{f_{y}}$$

where *FT_normalized_* is the normalized flexural toughness (kN∙mm), *f_y,HSC_* is the yield strength of the rebar used in the HSC series beams (MPa), and *f_y_* is the yield strength of rebar used in test beams (MPa).

**Figure 20:**
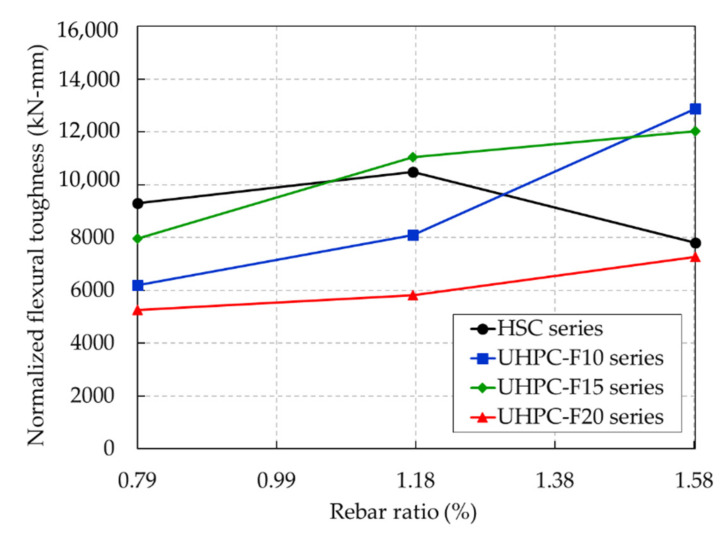
Normalized toughnesses of the UHPC and HSC beams.

The normalized flexural toughness results are given in Table 5 and are also shown in Figure 20. These results show that the normalized flexural toughness of the UHPC and HSC beams was affected by increasing the rebar ratio.

The normalized flexural toughness of the HSC beams increased slightly with rebar ratios from 0.79% to 1.18% and then showed a dramatic decrease with rebar ratios from 1.18% to 1.58%. For a rebar ratio of 0.79% (R1), the HSC beam exhibited greater normalized flexural toughness than any other UHPC beams with the same rebar ratio. The normalized flexural toughness of the HSC-R1 beam was 1.5, 1.2, and 1.8 times greater than that of the UHPC-F10-R1, UHPC-F15-R1, and UHPC-F20-R1 beams, respectively. For a rebar ratio of 1.18%, the normalized flexural toughness of the HSC-R2 beam was greater than that of the UHPC-F10-R2 and UHPC-F20-R2 beams. Moreover, compared to the UHPC-F15-R2 beam, the HSC-R2 beam exhibited a lower normalized flexural toughness. Consequently, the normalized flexural toughness of the UHPC-F10-R3 and UHPC-F15-R3 beams was 1.6 and 1.5 times greater than that of the HSC-R3 beam. In addition, for the HSC beam, the normalized flexural toughness at a rebar ratio of 1.58% (R3) was 16% less than that at a rebar ratio of 0.79% (R1). This finding implies that at higher rebar ratios, the normalized flexural toughness of the UHPC beams was greater than that of the HSC beams.

Furthermore, the normalized flexural toughness of the UHPC beam series exhibited a significant increase with increasing rebar ratio. In particular, the normalized flexural toughness of the UHPC-F10 beam series was improved considerably at rebar ratios of 1.18% to 1.58%. The lowest normalized flexural toughness was obtained from the UHPC-F20 beam series. This finding implies that the steel fiber content of 2.0% in the UHPC beam series in this study had an adverse effect on the distribution and orientation of steel fibers, thereby reducing the flexural toughness of the UHPC beams. However, regardless of the lowest normalized flexural toughness of the UHPC-F20 beam series, the gradual increasing tendency of the normalized flexural toughness with the increase in rebar ratio was also exhibited in this series.

**Figure 21:**
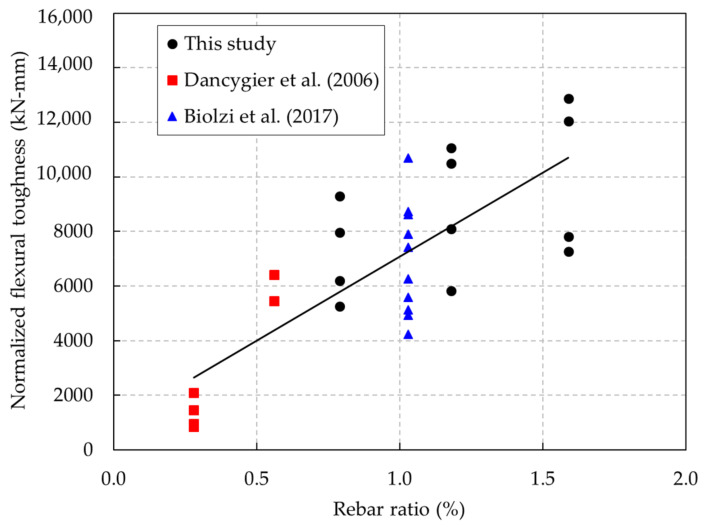
Comparison of the normalized toughness in this study and in previous studies.

A comparison between the normalized flexural toughness of this study and that from the previous research [16,36] was performed, as shown in Figure 21. This comparison indicates that the overall normalized flexural toughness values of the beams were improved as the rebar ratio increased. It also implies that the experimental result tendency of the normalized toughness in this study had good agreement with that in the previous research.

## 5. Conclusions

This study investigated the flexural behavior of UHPC beams with various rebar ratios and steel fiber volume contents. Comparisons between UHPC and HSC beams regarding the cracking pattern, failure, load-deflection behavior, ductility, and flexural toughness were presented. Based on the experimental investigations, the following conclusions can be drawn:

(1) Because of the bridging effect provided by steel fibers, the tensile stress in the UHPC beams was carried across the cracks and transferred into the surrounding matrix. Consequently, multiple microcracks with tight spaces were observed in the UHPC beams, and these beams failed by the pullout of the steel fibers at the major crack.
(2) At the same rebar ratio, the UHPC beams exhibited higher flexural capacity than the HSC beams. In addition, the ultimate deflection of the UHPC beams was affected not only by the rebar ratio but also by the steel fiber contents, whereas the ultimate deflection of the HSC beams decreased as the rebar ratio increased.
(3) The addition of steel fibers in the UHPC beams improved the energy absorption capacity of the beams. The test results indicated that a substantial amount of energy was released after the steel fibers were pulled out of the matrix, which resulted in localized cracking and subsequently beam failure.
(4) After initial cracking, the slopes of the moment-curvature curves of the UHPC beams were steeper than those of the HSC beams. Therefore, the presence of steel fibers in the UHPC matrix prevented the widening of the cracks and contributed to the development of flexural rigidity of the UHPC beams.
5) The ductility index of the UHPC beam was much smaller than that of the HSC beams at a rebar ratio of 0.79%. However, overall, the ductility index of the UHPC beams was greater than that of the HSC beams at a rebar ratio of 1.58%. This finding means that the presence of steel fibers could predominantly affect the ductility of the UHPC beams at a low rebar ratio.
6) The normalized flexural toughness of the UHPC beams was improved significantly by increasing the rebar ratios and steel fiber contents. However, the addition of a steel fiber content of 2.0% had a negative effect on the flexural toughness of the UHPC beams. Furthermore, overall, the normalized flexural toughness of the HSC beams decreased as the rebar ratio increased.

## Figures and Tables

**Table 1:** Mixing proportions.

Mixture	W/B	Unit Content (kg/m^3^)								Steel Fiber by Concrete Volume
		W	OPC	BFS	SF	Zr	S	F	G	(%)
HSC	0.15	150.0	700.0	150.0	150.0	-	467.5	-	765.1	-
UHPC-F10	0.22	209.0	770.0	135.0	-	58.0	847.0	231.0	-	1.0
UHPC-F15	0.22	209.0	770.0	135.0	-	58.0	847.0	231.0	-	1.5
UHPC-F20	0.22	209.0	770.0	135.0	-	58.0	847.0	231.0	-	2.0

W: water; B: binders; OPC: ordinary Portland cement; BFS: blast furnace slag; SF: silica fume; Zr: zirconium powder; S: sand; F: filler; and G: coarse aggregate.

**Table 2:** Physical and chemical properties of binder materials.

Type	OPC	BFS	SF	Zr
Density (g/cm^3^)	3.15	2.91	2.10	2.50
Surface area (cm^2^/g)	3413	4463	240,000	85,800
SiO_2_ (%)	21.01	34.56	96.00	94.00
Al_2_O_3_ (%)	6.40	14.78	0.25	0.22
Fe_2_O_3_ (%)	3.12	0.09	0.12	0.11
CaO (%)	61.33	41.32	0.38	0.50
MgO (%)	3.02	4.90	0.10	-
SO_3_ (%)	2.14	2.78	-	-
Ignition loss (%)	1.40	0.05	1.50	0.10

**Table 3:** Details of the beams.

Beam Specimen	Concrete Type	Beam Section		Concrete						Rebar				
		Width	Height	Compressive Strength	S.D.	Elastic Modulus	S.D.	Tensile Strength	S.D.	Nominal Diameter	Number of Rebars	Yield Strength	Rebar Area	Rebar Ratio
		b	h	$f_{c}^{'}$		$E_{c}$		$f_{t}$				*f_y_*	*A_s_*	*ρ*
		(mm)	(mm)	(MPa)	(MPa)	(GPa)	(GPa)	(MPa)	(MPa)	(mm)		(MPa)	(mm^2^)	(%)
HSC-R1	HSC	200	300	125	5	43.3	1.3	-	-	16	2	420.8	397.2	0.79
HSC-R2	HSC	200	300							16	3	420.8	595.8	1.18
HSC-R3	HSC	200	300							16	4	420.8	794.4	1.58
UHPC-F10-R1	UHPC	200	300	125	7	41.5	2.1	4.7	0.7	16	2	459.4	397.2	0.79
UHPC-F10-R2	UHPC	200	300							16	3	459.4	595.8	1.18
UHPC-F10-R3	UHPC	200	300							16	4	459.4	794.4	1.58
UHPC-F15-R1	UHPC	200	300	138	4	41.9	1.8	8.7	1.3	16	2	459.4	397.2	0.79
UHPC-F15-R2	UHPC	200	300							16	3	459.4	595.8	1.18
UHPC-F15-R3	UHPC	200	300							16	4	459.4	794.4	1.58
UHPC-F20-R1	UHPC	200	300	140	5	43.5	0.7	9.1	2.8	16	2	459.4	397.2	0.79
UHPC-F20-R2	UHPC	200	300							16	3	459.4	595.8	1.18
UHPC-F20-R3	UHPC	200	300							16	4	459.4	794.4	1.58

S.D., standard deviation.

**Table 4:** Test results of the beams.

Beam Specimen	Initial Cracking State			Yielding State			Peak State			Ultimate Sate			Ultimate Strain	Deflection Ductility Index $\mu_{\Delta }=\frac{\Delta_{u}}{\Delta_{y}}$	Curvature Ductility Index $\mu_{\varphi }=\frac{\varphi_{u}}{\varphi_{y}}$
	*P_cr_*	*M_cr_*	*Δ_cr_*	*P_y_*	*M_y_*	*Δ_y_*	*P_p_*	*M_p_*	*Δ_p_*	*P_u_*	*M_u_*	*Δ_u_*	*ɛ_u_*		
	(kN)	(kN∙m)	(mm)	(kN)	(kN∙m)	(mm)	(kN)	(kN∙m)	(mm)	(kN)	(kN∙m)	(mm)	(μɛ)		
HSC-R1	21.1	12.7	0.7	69.1	41.5	10.1	88.6	53.2	119.3	88.2	52.9	120.7	3607	12.0	6.3
HSC-R2	25.3	15.2	1.0	110.2	66.1	18.1	123.8	74.3	99.6	123.3	74.0	96.5	3580	5.3	2.4
HSC-R3	28.6	17.2	0.6	141.0	84.4	15.1	154.5	92.7	56.2	152.3	91.4	62.5	3051	4.1	1.7
UHPC-F10-R1	64.8	38.9	2.1	135.1	81.1	11.4	137.6	82.6	12.7	93.2	55.9	67.0	1372	5.9	2.2
UHPC-F10-R2	65.1	39.1	2.1	180.4	108.2	14.8	181.8	109.1	18.7	149.1	89.5	61.8	1288	4.2	1.1
UHPC-F10-R3	66.3	39.8	2.0	220.2	132.1	15.0	223.7	134.2	18.5	179.2	107.5	79.2	744	5.3	1.0
UHPC-F15-R1	69.4	41.6	2.1	184.8	110.9	12.6	187.5	112.5	13.4	102.3	61.4	86.7	2200	6.9	3.0
UHPC-F15-R2	70.9	42.5	2.1	223.6	134.2	15.0	224.4	134.6	15.5	100.0	60.0	90.8	1260	6.1	1.0
UHPC-F15-R3	63.0	37.8	1.8	233.9	140.4	15.3	243.3	146.0	19.4	191.7	115.0	69.3	1941	4.5	1.8
UHPC-F20-R1	91.1	54.7	2.9	166.6	100.0	10.8	167.2	100.3	11.0	89.6	53.8	52.6	1650	4.9	1.7
UHPC-F20-R2	91.0	54.6	3.1	212.2	127.3	13.7	217.5	130.5	14.7	157.9	94.7	41.5	1600	3.0	1.2
UHPC-F20-R3	87.0	52.2	2.9	238.5	143.1	16.0	239.4	143.6	16.4	193.4	116.0	46.3	2029	2.9	1.8

*P_cr_, M_cr_, Δ_cr_*: the load, moment, and deflection at the initial cracking state, respectively. *P_y_, M_y_, Δ_y_*: the load, moment, and deflection at the yielding state of rebar, respectively. *P_p_, M_p_, Δ_p_*: the load, moment, and deflection at the peak load state, respectively. *P_u_, M_u_, Δ_u_*: the load, moment, and deflection at the ultimate state, respectively. *ɛ_u_*: the strain of concrete at the ultimate state. *φ_y_*: the curvature at the yielding state of rebar. *φ_u_*: the curvature at the ultimate state.

**Table 5:** Flexural toughness of the beams.

Beam Specimen	Flexural Toughness (kN∙mm)	Rebar yield Strength (MPa)	Normalized Flexural Toughness (kN∙mm)	Remarks
HSC-R1	9300	420.8	9300	Reference beams
HSC-R2	10,491	420.8	10,491	
HSC-R3	7817	420.8	7817	
UHPC-F10-R1	6759	459.4	6191	Normalized beams
UHPC-F10-R2	8845	459.4	8102	
UHPC-F10-R3	14,057	459.4	12,876	
UHPC-F15-R1	8692	459.4	7961	
UHPC-F15-R2	12,068	459.4	11,054	
UHPC-F15-R3	13,143	459.4	12,039	
UHPC-F20-R1	5732	459.4	5251	
UHPC-F20-R2	6356	459.4	5822	
UHPC-F20-R3	7938	459.4	7271	
